# Comparison of NH_3_ and N_2_O Plasma Treatments on Bi_2_O_3_ Sensing Membranes Applied in an Electrolyte–Insulator–Semiconductor Structure

**DOI:** 10.3390/membranes12020188

**Published:** 2022-02-05

**Authors:** Chyuan-Haur Kao, Kuan-Lin Chen, Yi-Shiang Chiu, Lin Sang Hao, Shih-Ming Chen, Ming-Hsien Li, Ming-Ling Lee, Hsiang Chen

**Affiliations:** 1 Department of Electronic Engineering, Chang Gung University, 259 Wen-Hwa 1st Road, Kwei-Shan District, Taoyuan City 333, Taiwan; chkao@mail.cgu.edu.tw (C.-H.K.); chkao@mail.cgu.edu (K.-L.C.); 2 Kidney Research Center, Department of Nephrology, Chang Gung Memorial Hospital, Chang Gung University, No. 5 Fuxing Street, Guishan District, Taoyuan City 333, Taiwan; 3 Department of Electronic Engineering, Ming Chi University of Technology, 284 Gungjuan Road, Taishan District, New Taipei City 243, Taiwan; 4 Department of Applied Materials and Optoelectronic Engineering, National Chi Nan University, Puli, Nantou 545, Taiwan; s109328018@mail1.ncnu.edu.tw (Y.-S.C.); s109328023@mail1.ncnu.edu.tw (L.S.H.); s107328009@mail1.ncnu.edu.tw (S.-M.C.); mhli1125@ncnu.edu.tw (M.-H.L.); 5 Department of Electro-Optical Engineering, Minghsin University of Science and Technology, No. 1, Xinxing Road, Xinfeng, Hsinchu 30401, Taiwan

**Keywords:** bismuth trioxide, plasma treatment, NH_3_ and N_2_O, nitrogen passivation, grainization

## Abstract

In this study, bismuth trioxide (Bi_2_O_3_) membranes in an electrolyte–insulator–semiconductor (EIS) structure were fabricated with pH sensing capability. To optimize the sensing performance, the membranes were treated with two types of plasma—NH_3_ and N_2_O. To investigate the material property improvements, multiple material characterizations were conducted. Material analysis results indicate that plasma treatments with appropriate time could enhance the crystallization, remove the silicate and facilitate crystallizations. Owing to the material optimizations, the pH sensing capability could be greatly boosted. NH_3_ or N_2_O plasma treated-Bi_2_O_3_ membranes could reach the pH sensitivity around 60 mV/pH and show promise for future biomedical applications.

## 1. Introduction

Fifty years ago, the first ion-sensitive field-effect transistor (ISFET) was invented by Bergveld in 1970 [1,2]. Following the invention, semiconductor-based ion sensing technology [1,2,3] has been developed since the late 20th century. Among various types of ion sensing semiconductor devices, electrolyte–insulator–semiconductor (EIS) sensors with rapid response, high reliability and simple structure have been intensively studied [4]. Because of low capacitance and poor electrochemical properties, SiO_2_ has been replaced by various oxides to improve the membranes properties [5]. Recently, Ta_2_O_5_ [6], WO_3_ [7], and La_2_O_3_ [8] have emerged as novel membrane materials [9]. However, to further boost the membrane sensing performance, novel materials and new treatments are worthwhile to be explored. Bismuth trioxide (Bi_2_O_3_) with a bandgap around 2.5 eV has been utilized as photocatalyst [10], super capacitors, and gas sensor materials [11]. However, Bi_2_O_3_-based pH sensing membranes [12] have not been clearly reported, yet. Furthermore, to enhance the sensing capability, Bi_2_O_3_ membranes were treated with two types of plasma- NH_3_ and N_2_O [13,14]. To investigate the improvement of the treatment, multiple material analysis techniques including X-ray diffraction (XRD), X-ray photoelectron spectroscopy (XPS), atomic force microscopy (AFM), and secondary ion mass spectrometry (SIMS) were performed. Material analysis results indicate that Bi_2_O_3_ membranes treated with NH_3_ plasma for 3 min and N_2_O plasma for 1 min had strong crystallization, silicate suppression, high grainization, and effective nitrogen passivation. Moreover, the pH sensing measurements [15] indicate that Bi_2_O_3_ membranes treated in these plasma treatment conditions had high pH sensitivity around 60 mV/pH and high linearity close to 100%. Hysteresis and drift [16] rate evaluation also reveal that the membrane treated in these conditions had the lowest hysteresis voltage and the smallest drift rate. NH_3_ and N_2_O plasma treatments could incorporate the nitrogen atoms into the deep part of the devices to fix the defects and eliminate the silicates.

According to previous reports [17,18], plasma treatment can eliminate the silicate layer because, because silicates can have chemical reaction and transform from SiOx with dangling bonds to Si–O–Si bonds. The atoms in the plasma such as F or N can facilitate the combination of the silicon or oxygen dangling bonds to form well-crystallized Si–O–Si [19]. Therefore, silicate can be reduced, and near-perfect crystals can replace the silicate. The effective electric field across the membranes can be enhanced so the sensing capability can be improved [20,21]. Moreover, the nitrogen incorporation in the bulks may form NH_2_ bond to strengthen the chemical bonds and improve the material properties [22]. On the other hand, plasma treatment of the sensing film could cause an EIS structure sensitive to H^+^ ions [23] because the increase of the metal ions produced on the surface sites to decrease the diffusion capacitance in the solution and enhance the sensitivity. Furthermore, based on the Gouy–Chapman–Stern model [24,25], the sensing parameter β is proportional to the density of surface states, as shown in (1), where Ns is the number of surface sites per unit surface area and C_DL_ is the double layer capacitance. Therefore, the sensing capability can be enhanced by NH_3_ and N_2_O plasma treatments.

(1) $$\beta =\frac{2q^{2}\mathrm{Ns}\sqrt{K_{a}K_{b}}}{{\mathrm{KTC}}_{\mathrm{DL}}}$$

Owing to high sensitivity, fair linearity, stable response, Bi_2_O_3_-based EIS membranes [26] with NH_3_ or N_2_O plasma treatments show promise for future industrial biomedical sensing [27] applications.

## 2. Experimental

**Figure 1:**
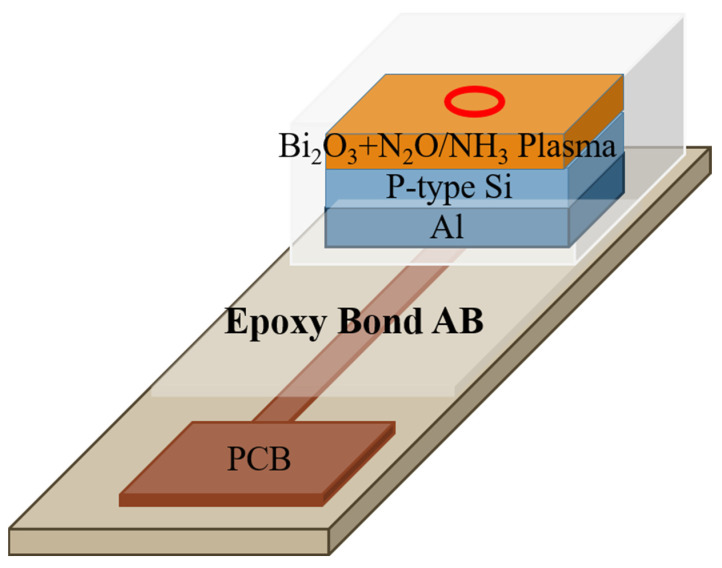
A Bi_2_O_3_-based membrane in an EIS structure with plasma treatments.

To prepare the EIS sensor with Bi_2_O_3_ sensing film, 3.95 g of bismuth nitrate (Bi(NO₃)₃·5H₂O) was dissolved in 20 mL of nitric acid with the solution concentration of 1 M. The sol-gel solution was dropped onto the cleansed p-Si substrate. Then, a Bi_2_O_3_ film formed on it, and then the plasma treatments were performed. The samples were treated with NH_3_ and N_2_O plasma at 100 W RF power and 500 mTorr processing pressure for 1 min, 3 min, and 6 min, respectively. Then, an Al film with a thickness of 300 nm was deposited on the back of the silicon wafer and the silicone glue was used to define the sensing window, and the device was glued to be fixed on a PCB board. Finally, AB glue was used for packaging to prevent oxidation. The device structure is illustrated in Figure 1.

## 3. Results and Discussion

**Figure 2:**
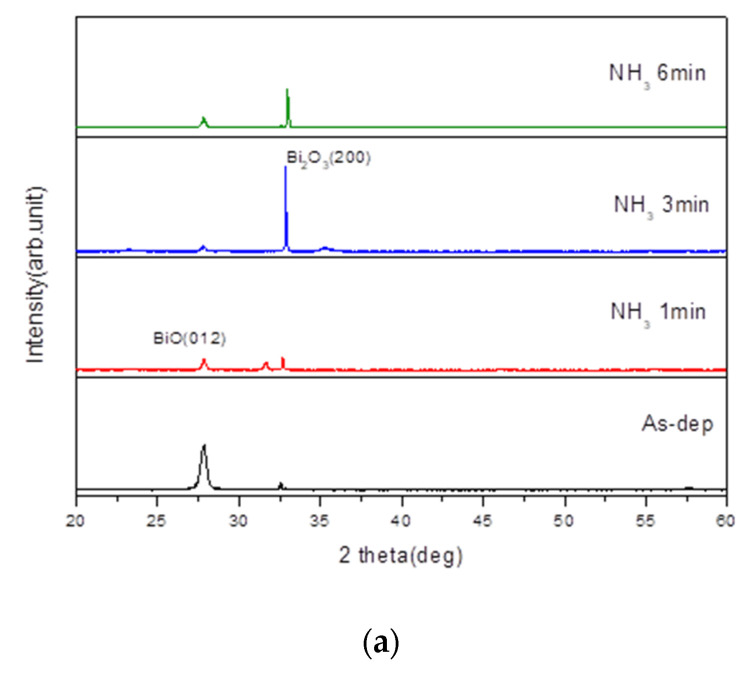

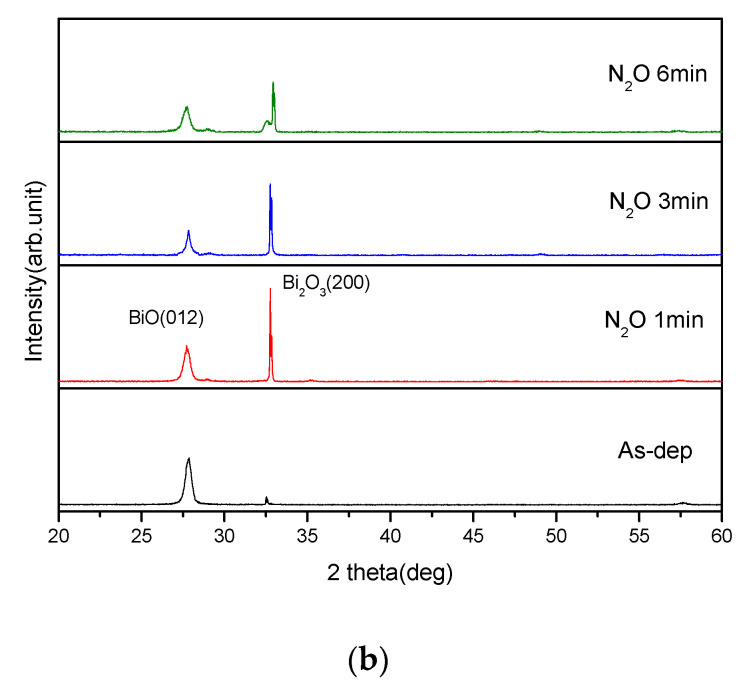
XRD patterns of the Bi_2_O_3_ film after (**a**) NH_3_ and (**b**) N_2_O plasma treatments for various time.

To examine the crystalline structures of the membranes, XRD was used. Figure 2a shows the X-ray diffraction patterns of Bi_2_O_3_ film after NH_3_ plasma treatments for various times. The two diffraction peaks BiO (012) and Bi_2_O_3_ (200) are located at 27.8° and 32.6°, respectively. The as-deposited sample shows a peak of BiO. After NH_3_ plasma treatment for 3 min, the sample shows the strongest peak of Bi_2_O_3_ (200) among all the samples.

By contrast, Figure 2b shows the X-ray diffraction analysis of the Bi_2_O_3_ film after N_2_O plasma treatments for various times. The diffraction peaks BiO (012) and Bi_2_O_3_ (200) are located at 27.8° and 32.6°, respectively. The as-deposited sample also shows a peak of BiO. After N_2_O plasma treatment for 1 min, the sample shows the strongest Bi_2_O_3_ (200) peak among all the samples. With the increase of the plasma treatment time, the intensity of the Bi_2_O_3_ (200) peak gradually decreased.

**Figure 3:**
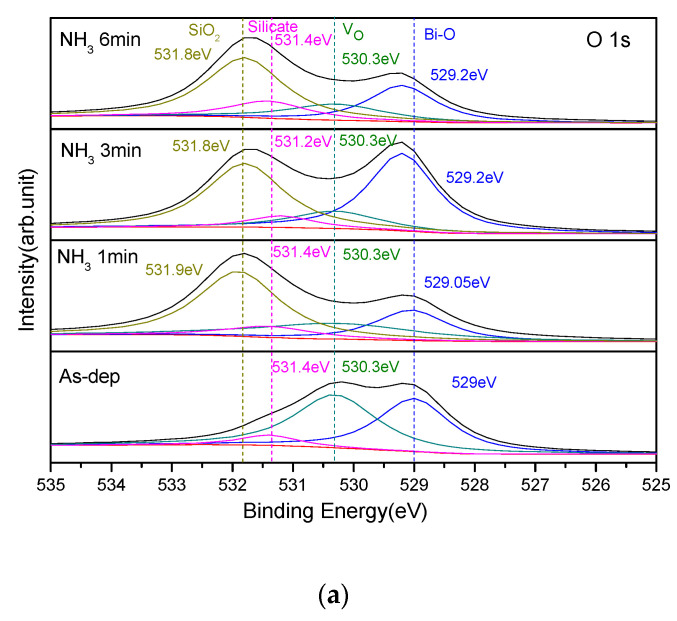

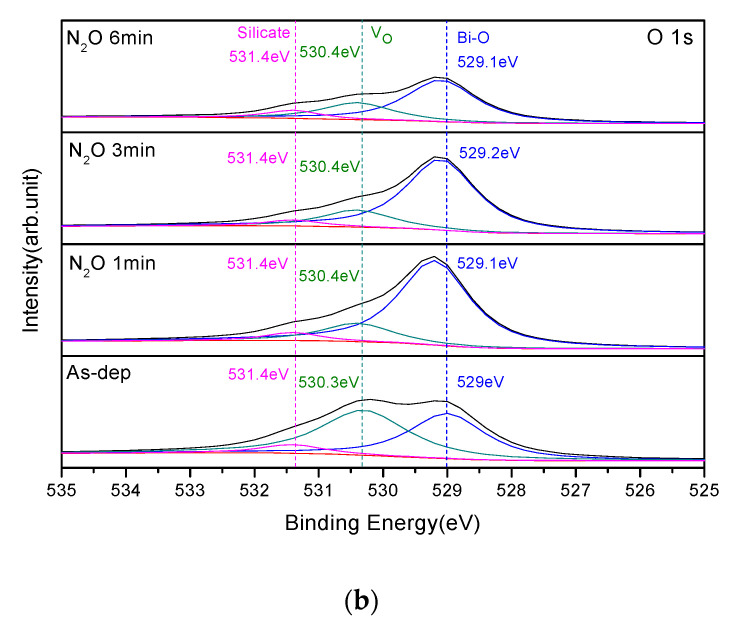
The O1s XPS spectra of the Bi_2_O_3_ film after (**a**) NH_3_ and (**b**) N_2_O plasma for various treatment time.

Furthermore, XPS analysis was used to study the chemical bonding state of the Bi_2_O_3_ sensor film after NH_3_ and N_2_O plasma treatments. The O1s spectra of the samples after NH_3_ plasma treatments is shown in Figure 3a. The as-deposited and annealed samples have 4 peak fitting curves, namely SiO_2_ (531.8 eV), silicate (531.4 eV), oxygen defect (530.3 eV), and Bi-O (529 eV). After the NH_3_ plasma treatment, the oxygen defects and slicates were significantly reduced. Because NH_3_ plasma treatment could dope N into the film to improve dangling bonds and strain bonds, the sensing properties were improved.

The O1s spectra of the samples after N_2_O plasma treatment is shown in Figure 3b. The as-deposited and annealed samples have three peak fitting curves, namely, silicate (531.4 eV), oxygen defect (530.3 eV), and Bi-O (529 eV). After N_2_O plasma treatment, the oxygen defects and silicates were significantly suppressed. Since N_2_O plasma treatment could incorporate N into the film, mitigate dangling bonds and strain bonds, and strengthen the film structure, the sensing behaviors could be improved. Results indicate that N atoms in the plasma could transform SiO_x_ with dangling bonds to form well-crystallized Si–O–Si.

**Figure 4:**
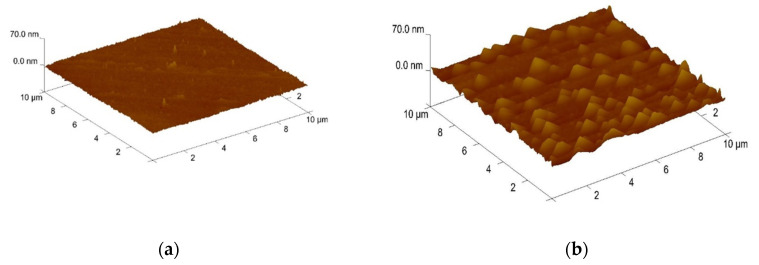

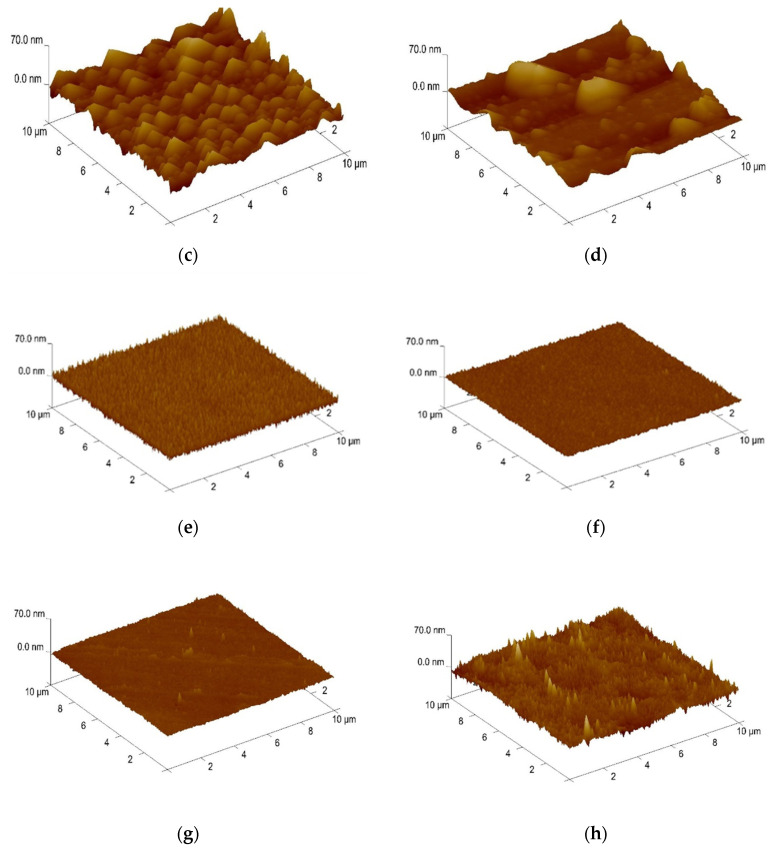
Three-dimensional (3D)-AFM images of Bi_2_O_3_ film after NH_3_ plasma treatment for (**a**) 0 min RMS:1.31 nm; (**b**) 1 min NH_3_ plasma RMS: 5.32 nm; (**c**) 3 min NH_3_ plasma RMS: 15.56 nm; (**d**) 6 min NH_3_ plasma RMS: 11.33 nm. Three-dimensional (3D)-AFM of Bi_2_O_3_ film after different N_2_O plasma treatment for (**e**) 0 min RMS:1.31 nm; (**f**) 1 min N_2_O plasma RMS: 3.9 nm; (**g**) 3 min N_2_O plasma RMS: 3.83 nm; (**h**) 6 min N_2_O plasma RMS: 3.21 nm.

Figure 4a–d shows the atomic force microscope (AFM) images of the Bi_2_O_3_ film after NH_3_ plasma treatments for various times, and Figure 4e–h shows the atomic force microscope (AFM) images of the Bi_2_O_3_ film after N_2_O plasma treatments for various times. The root mean square (Rms) roughness of the sample without plasma treatment and of the samples after NH_3_ plasma treatment for 1, 3, and 6 min were 1.31, 5.32, 15.56, and 11.33 nm, respectively. The root mean square (Rms) roughness of the sample without plasma treatment and of the samples after N_2_O plasma treatment for 1, 3, and 6 min were 1.31, 3.9, 3.83, and 3.21 nm, respectively. After 3 min of NH_3_ plasma treatment of 1 min of N_2_O plasma treatment, the Bi_2_O_3_ film has the largest Rms value. The incorporation of N can passivate the defects improve the crystalline structure, and strengthen the grainization, thereby increasing the surface sites and improving the sensing characteristics.

**Figure 5:**
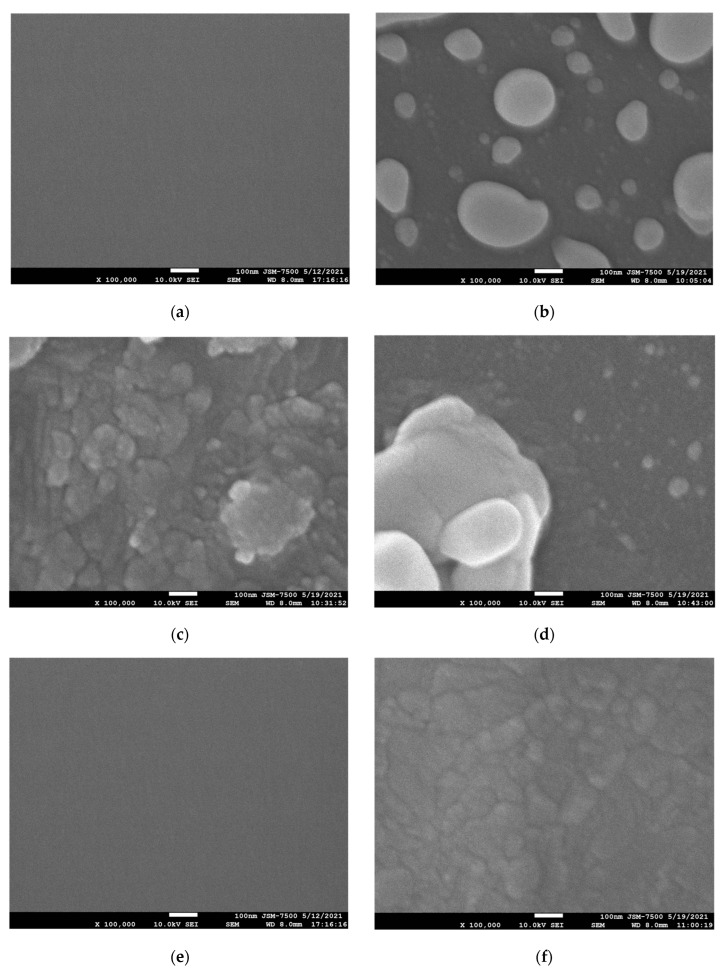
FESEM of Bi_2_O_3_ film after different NH_3_ and N_2_O plasma treatment times: (**a**) As-dep; (**b**) 1 min NH_3_ plasma; (**c**) 3 min NH_3_ plasma; (**d**) 6 min NH_3_ plasma; (**e**) As-dep; (**f**) 1 min N_2_O plasma.

To examine the surface morphologies, Figure 5a–f shows the field emission scanning electron microscope (FESEM) images of the deposited Bi_2_O_3_ film and the Bi_2_O_3_ film after NH_3_ and N_2_O plasma treatments. After 1 min of NH_3_ plasma treatment, irregular crystals with uneven distribution were produced on the surface. After 3 min of plasma treatment, the crystals became denser. After 6 min of plasma treatment, the crystals became sparsely distributed again. Therefore, the NH_3_ plasma treatment in 3 min had the best material properties. (FESEM) images of the deposited Bi_2_O_3_ film and the Bi_2_O_3_ film before and after N_2_O plasma treatment for 1 min are shown in Figure 5e,f. After N_2_O plasma treatments for 1 min, the crystallization of the film became obvious. Due to the incorporation of N, the dangling bonds and strain bonds in the film can be fixed, and the crystalline structure could be strengthened, so the sensing could be improved.

In addition, the images of the two types of plasma treatments are compared. It can be found that the uniformity of the film after N_2_O plasma treatments is more uniform than that of the Bi_2_O_3_ film after NH_3_ plasma treatment. Therefore, Bi_2_O_3_ film maybe more stable after N_2_O plasma treatments than NH_3_ plasma treatments.

**Figure 6:**
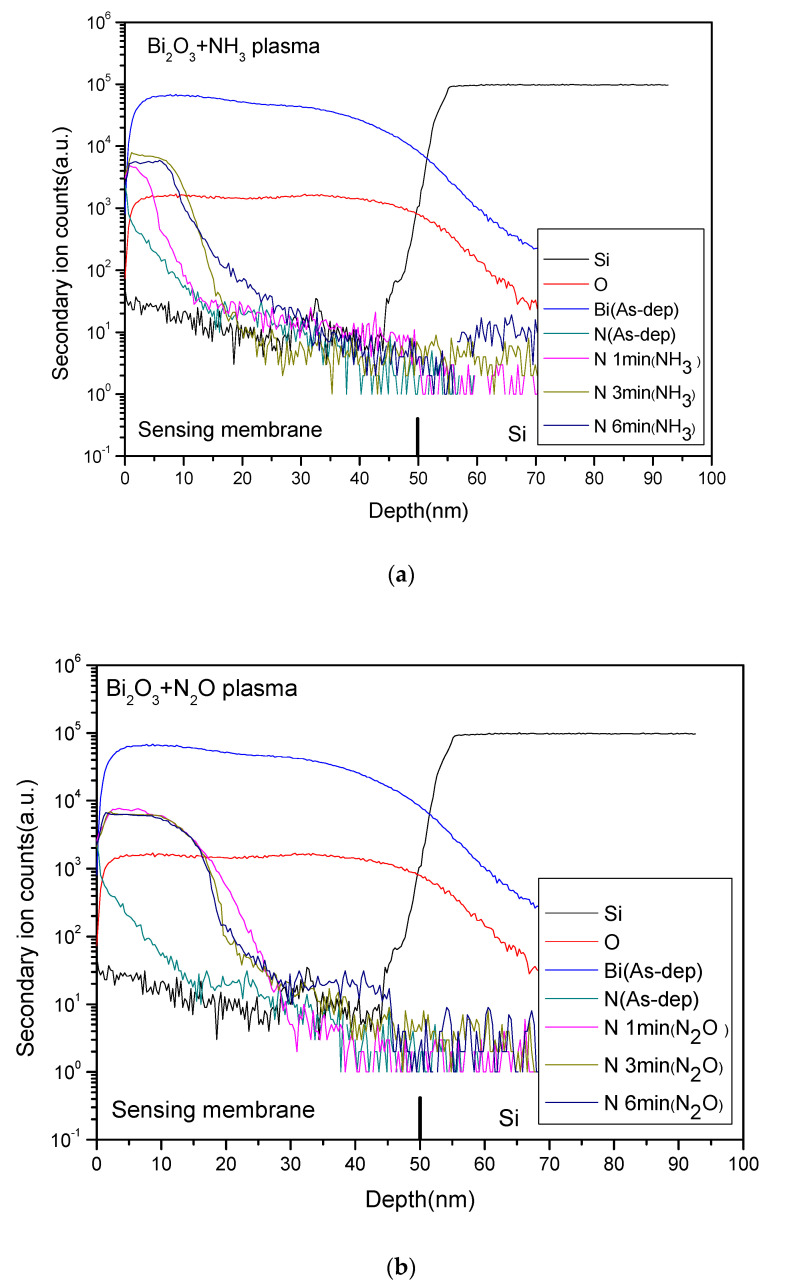
SIMS analysis for Bi_2_O_3_ film after: (**a**) NH_3_ plasma treatments; (**b**) N_2_O plasma treatments for various time.

Figure 6a,b shows the SIMS of the samples with NH_3_ and N_2_O plasma treatment for various time. It can be seen that as the plasma treatment time increased, the thickness of the sensing film decreased. As the film after plasma treatment would be etched with the increase of the plasma treatment time, the sensing characteristics were slightly reduced. On the other hand, nitrogen atoms can be introduced into the Bi_2_O_3_/Si interface by NH_3_ and N_2_O plasma treatment as shown in the two SIMS profiles. These accumulated nitrogen atoms can passivate the defects of the interface. Since the incorporation of N can improve the dangling bonds and strain bonds of the film, the sensing performance is improved.

As the two plasma treatments are compared, it can be found that the amount of nitrogen incorporated after the N_2_O plasma treatment was relatively stable, and the nitrogen content only slightly decreased with the increase of the plasma time. Therefore, the N_2_O plasma treatment had relatively stable sensing characteristics.

**Figure 7:**
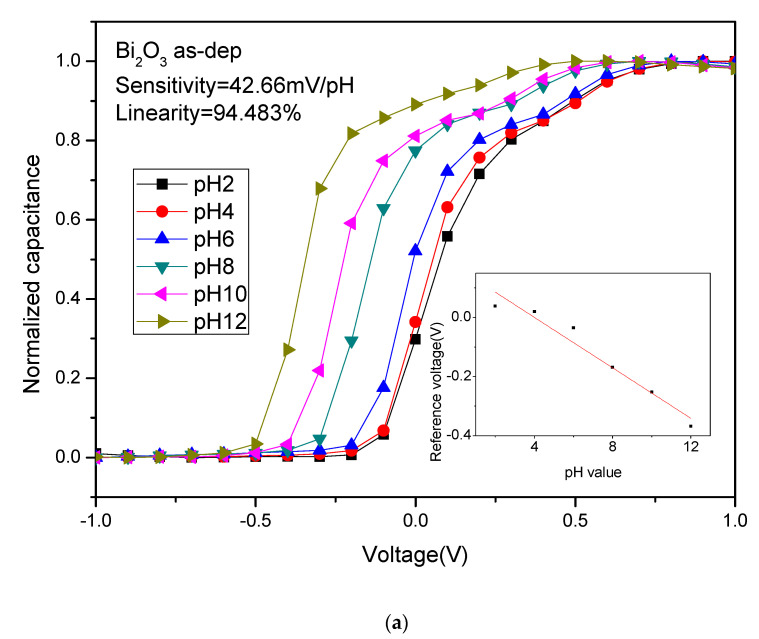

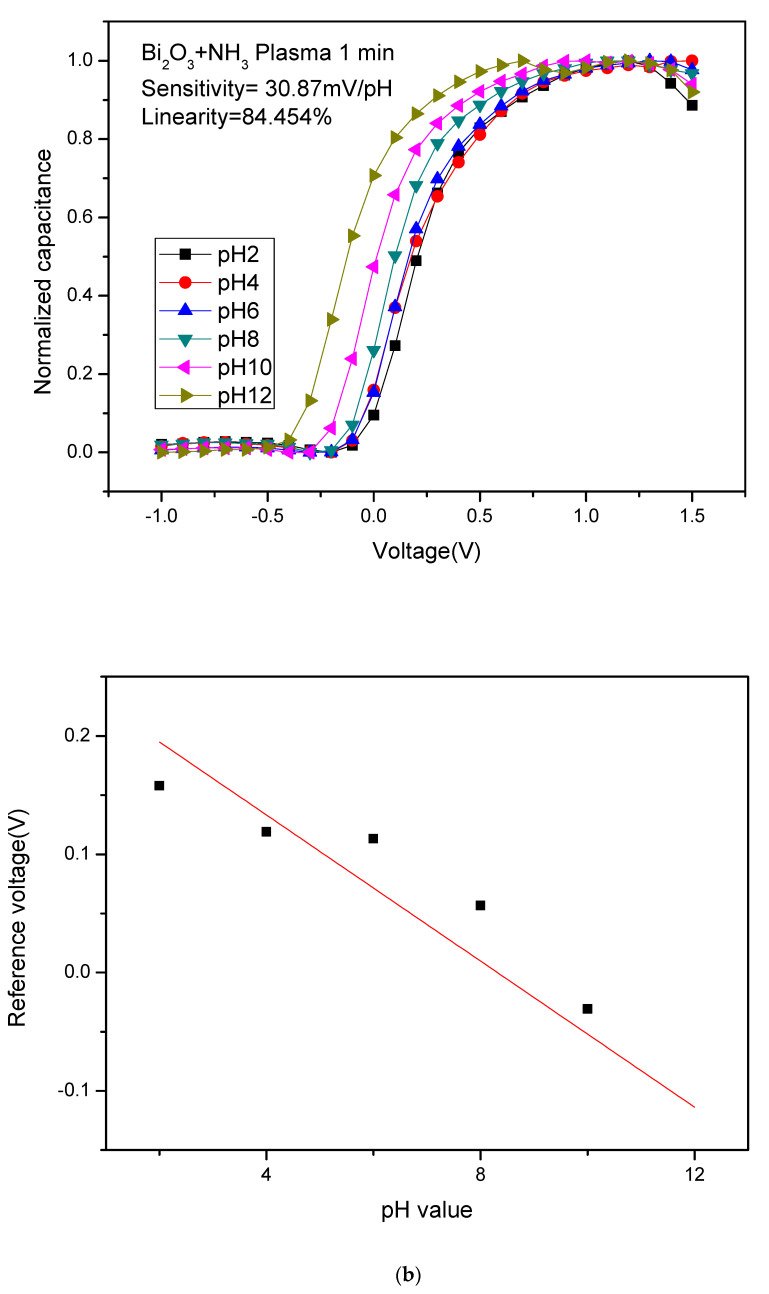

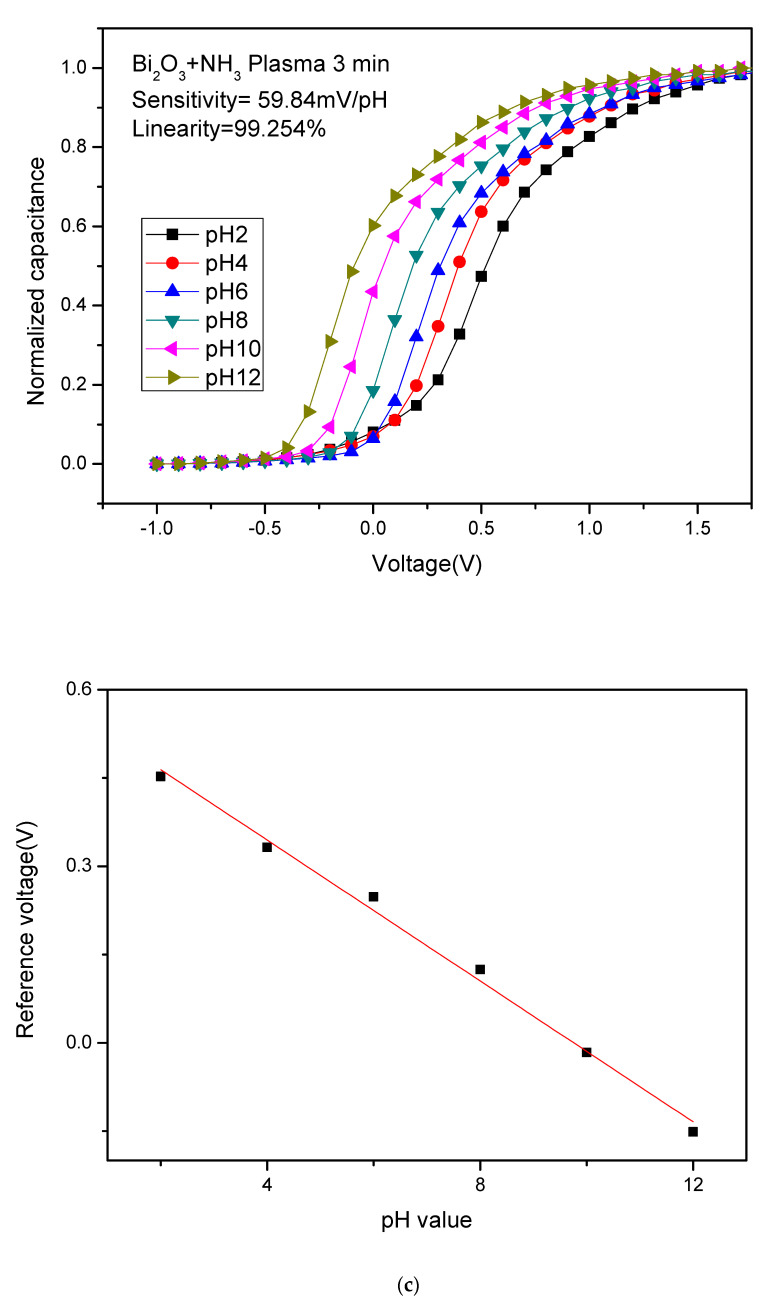

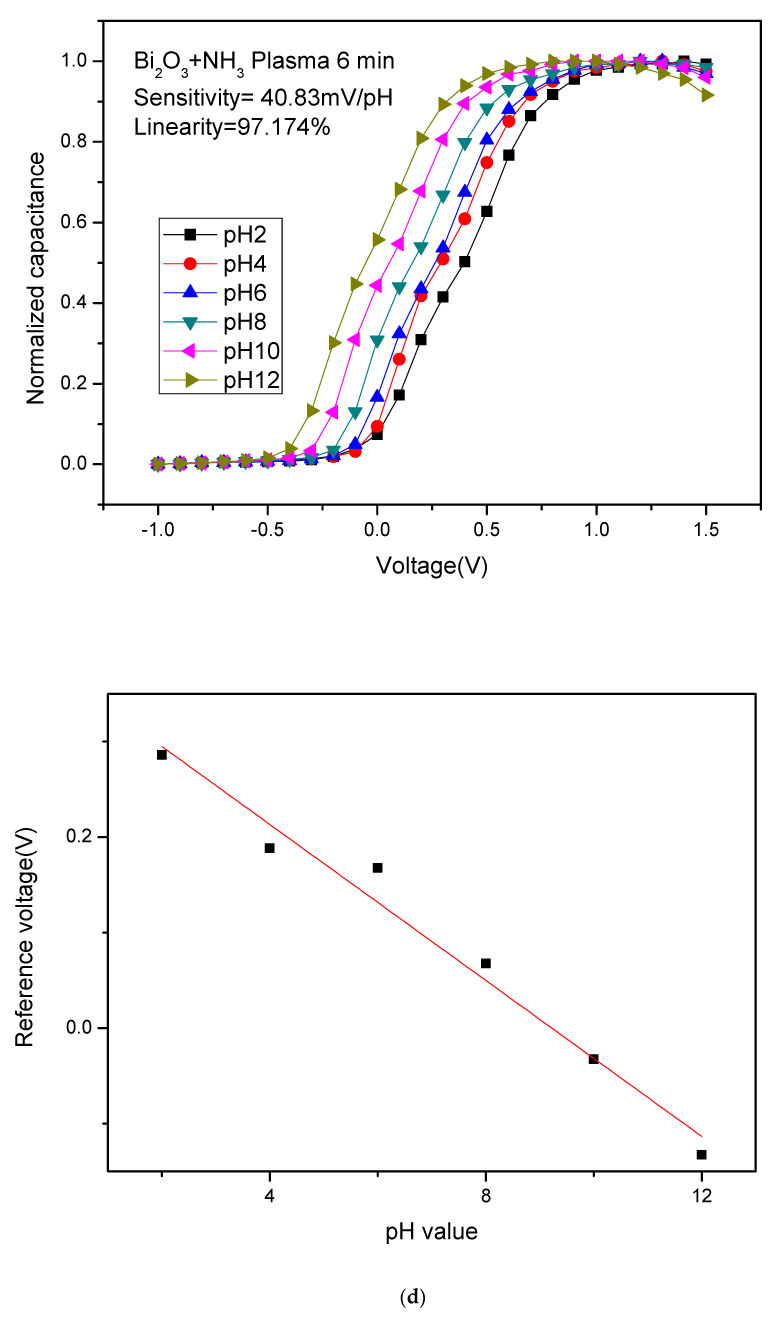

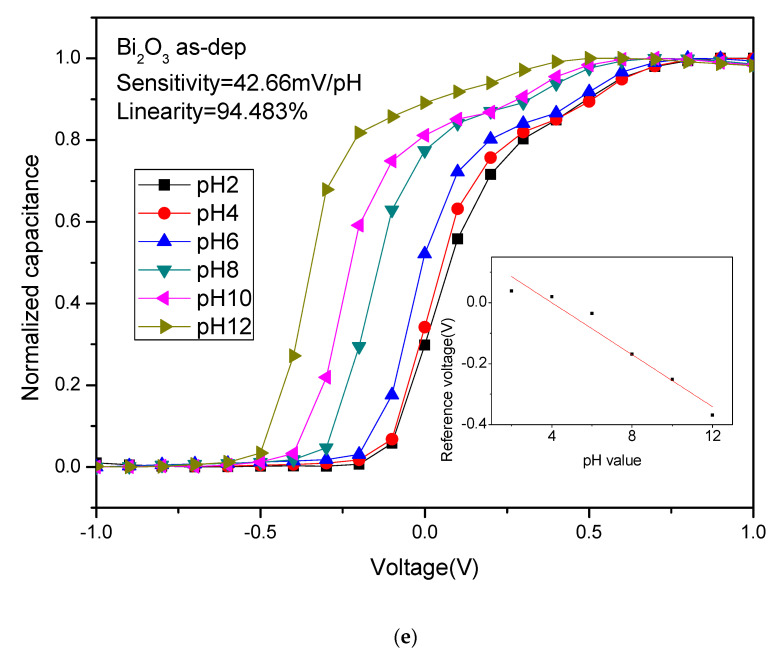

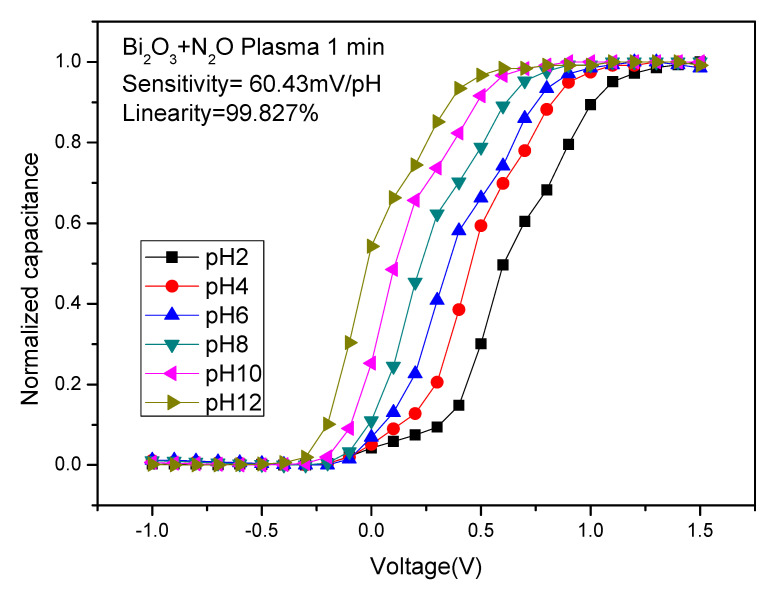

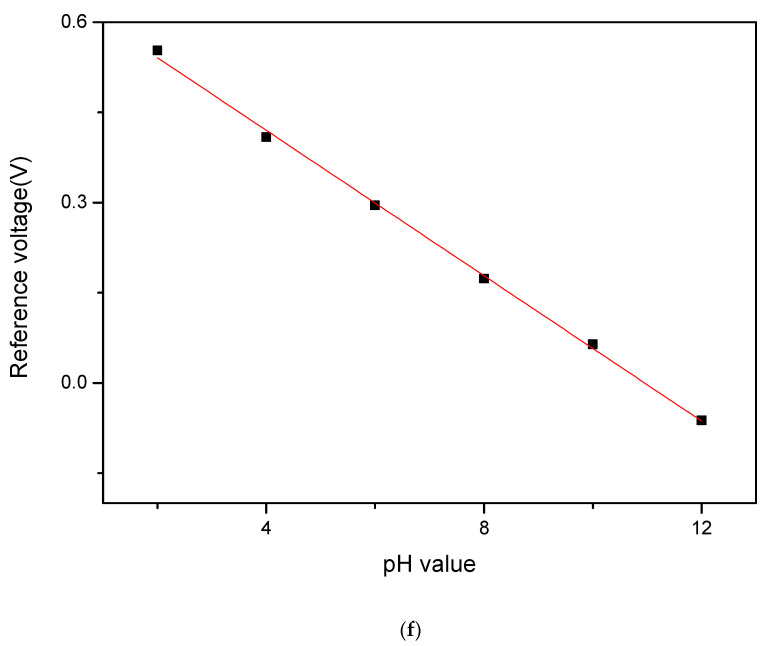

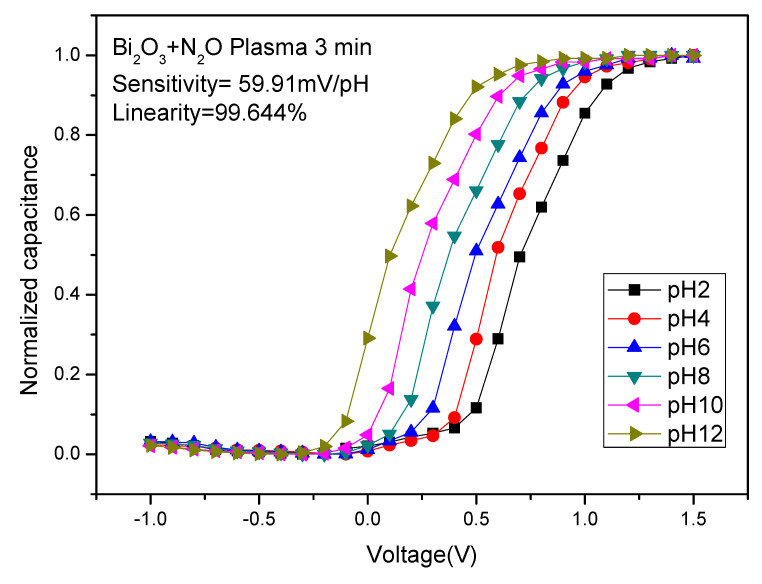

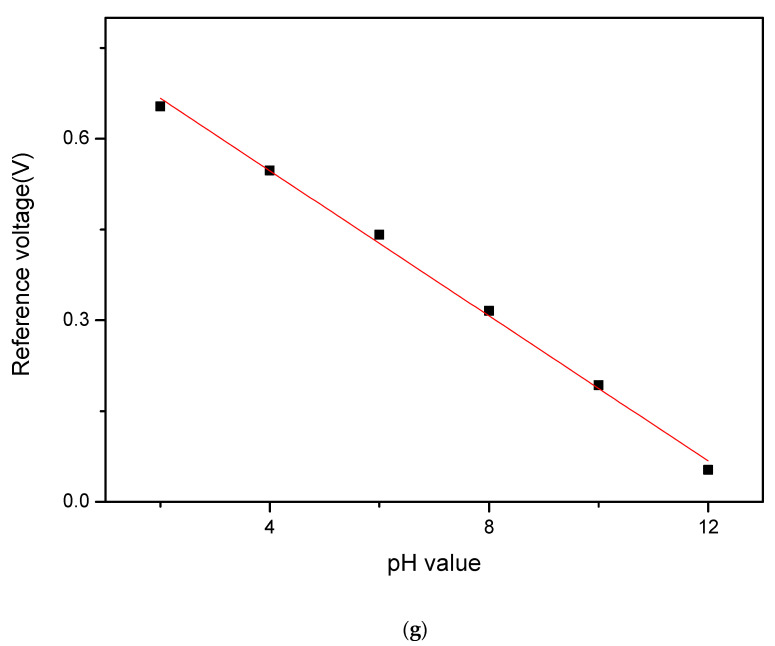

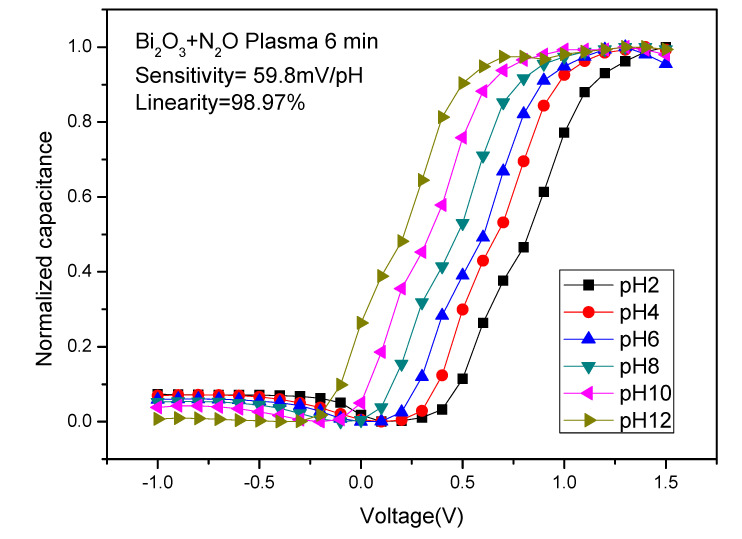

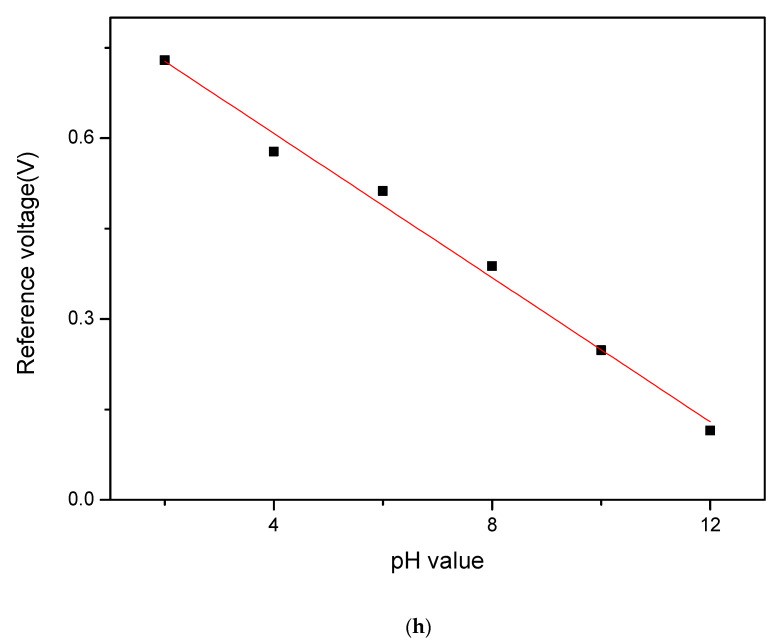
C–V curves of Bi_2_O_3_ sensing membrane with NH_3_ plasma for (**a**) as-dep, (**b**) 1 min, (**c**) 3 min, (**d**) 6 min NH_3_ plasma treatment. C–V curves of the Bi_2_O_3_ sensing membrane with N_2_O plasma for (**e**) as-dep, (**f**) 1 min, (**g**) 3 min, (**h**) 6 min N_2_O plasma treatment.

To assess the pH sensing behaviors, C–V curves of Bi_2_O_3_ after different NH_3_ and N_2_O plasma treatment conditions were measured. Figure 7a–d shows C–V curves of Bi_2_O_3_ after NH_3_ plasma for various times. The sensitivity value without NH_3_ plasma treatment was 42.66 mV/pH, and the linearity was 94.483%. After NH_3_ plasma treatment for 1 min, 3 min and 6 min, the sensitivity values became 30.87, 59.84 and 40.83 mV/pH, respectively, and the linearity values became 84.45%, 99.25% and 97.17%. As for the sensitivity among the Bi_2_O_3_ samples with different NH_3_ plasma time, it can be found that the Bi_2_O_3_ sensor film had the highest sensitivity after NH_3_ plasma treatment for the 3 min sample. Consistent with the FESEM images, the film treated in this condition produced densely arranged and layered crystals, which produced a larger contact area and increased sensitivity. After 3 min of NH_3_ plasma treatment, there were small and dense Bi_2_O crystals, thereby increasing the sensitivity of the sensing film.

Figure 7e–h shows the C–V curve of Bi_2_O_3_ after different N_2_O plasma treatment time. After N_2_O plasma treatment for 1 min, 3 min and 6 min, the sensitivity were 60.43, 59.91 and 59.8 mV/pH, respectively. The linearity was 99.82%, 98.97% and 99.64%. As for the sensitivity of Bi_2_O_3_ under different N_2_O plasma time, it can be found that the sensitivity of the Bi_2_O_3_ film after N_2_O plasma for various times and the samples in all the conditions were improved, consistent with FESEM images of the uniform distributed crystals under various plasma treatment conditions. After N_2_O plasma treatment, surface defects could be passivated, and dangling bonds and strain bonds can be fixed. Therefore, plasma treatments could improve the material properties and enhance the crystalline structure and grainization effect, and thereby increasing sensitivity.

**Figure 8:**
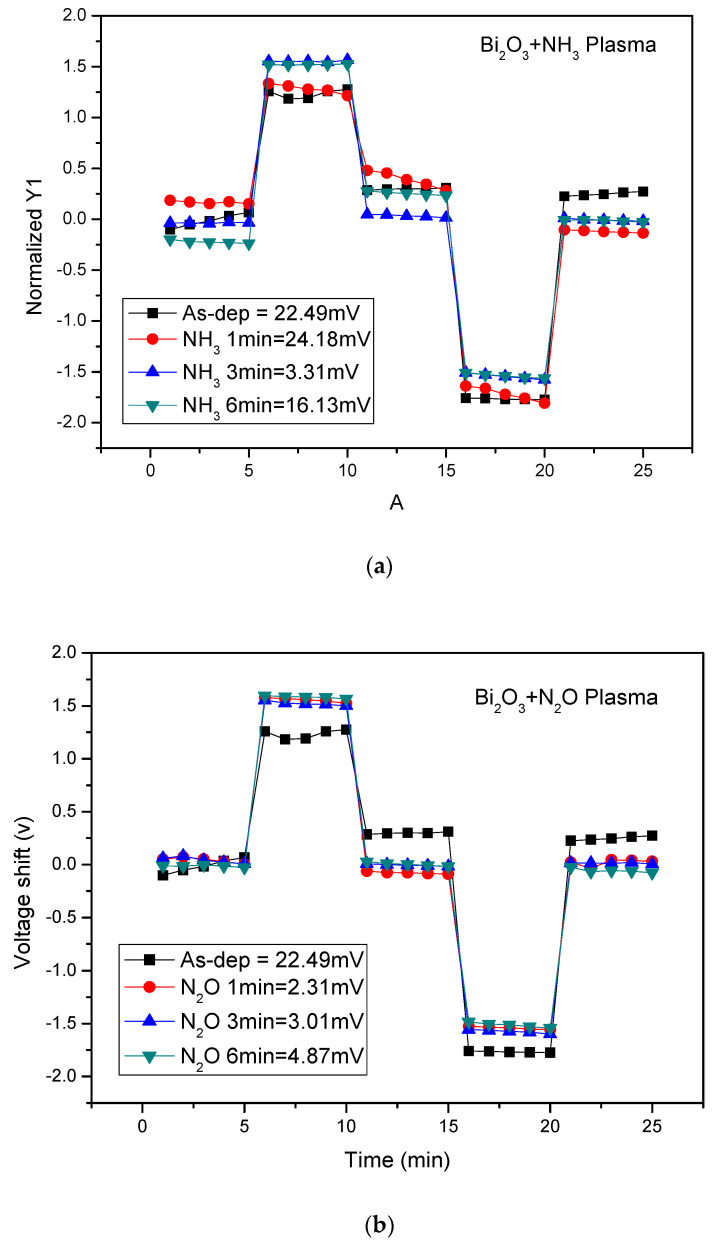

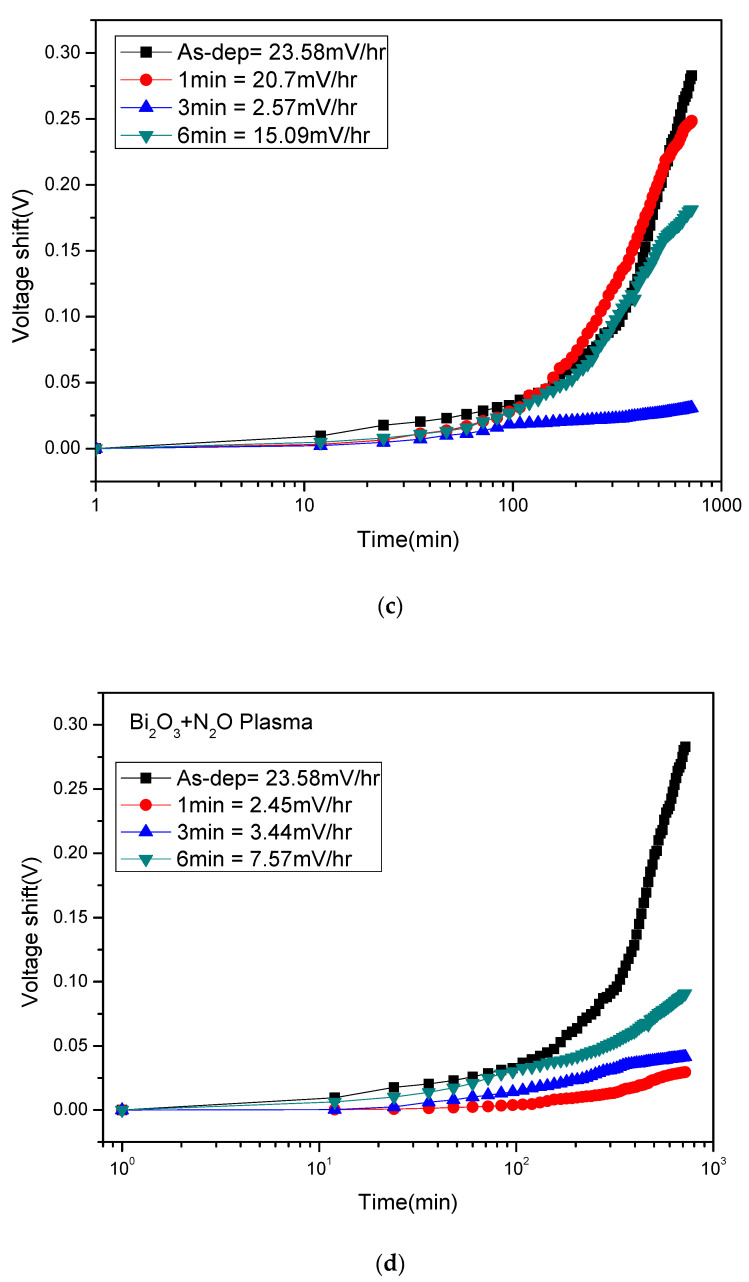
(**a**) Hysteresis voltage of the Bi_2_O_3_ sensing membrane after NH_3_ plasma treatment during the pH loop of 7→4→7→10→7. (**b**) Hysteresis voltage of the Bi_2_O_3_ sensing membrane after N_2_O plasma treatment during the pH loop of 7→4→7→10→7. (**c**) Drift voltage of the Bi_2_O_3_ sensing membrane after NH_3_ plasma treatment, then dipped in pH 7 buffer solution for 12 h. (**d**) Drift voltage of the Bi_2_O_3_ sensing membrane after N_2_O plasma treatment, then dipped in pH 7 buffer solution for 12 h.

To investigate the reliability of the membranes, Figure 8a shows the hysteresis voltage of the Bi_2_O_3_ sensing film after NH_3_ and N_2_O plasma treatment. The Bi_2_O_3_ sensing film without plasma had a hysteresis voltage of 22.49 mV, and the hysteresis voltage after 1, 3, and 6 min of NH_3_ plasma were 24.18, 3.31, and 16.13 mV, respectively. As for the Bi_2_O_3_ sensing film treated by NH_3_ plasma at different times, the NH_3_ plasma treatment for 3 min shows the lowest hysteresis voltage. Since the incorporation of N with NH_3_ plasma treatment can passivate the defects, thereby inhibiting the diffusion of reactive ions and delaying the reference voltage response. Combined with the XPS analysis, it can be seen that the 3 min NH_3_ plasma had the least oxygen vacancies, and the Bi-O bond was the strongest. After calculation, it can be known that the 3 min NH_3_ plasma has the lowest Bi^2+^ content, so the sensor film shows low hysteresis voltage.

The hysteresis voltage of the Bi_2_O_3_ sensing film after N_2_O plasma is shown in Figure 8b. The hysteresis voltage of the Bi_2_O_3_ sensing film without plasma was 22.49 mV, and the hysteresis voltage after 1, 3, and 6 min of N_2_O plasma were 2.31, 3.01, and 4.87 mV, respectively. It was observed that after 1 min of N_2_O plasma the membrane had a lower hysteresis voltage compared with all the other samples. As the plasma treatment time increased, the hysteresis voltage gradually increased because the crystals gradually became smaller, which caused the hysteresis voltage to rise.

Furthermore, Figure 8c,d shows the drift coefficient of the Bi_2_O_3_ sensing film after NH_3_ and N_2_O plasma, respectively. The drift coefficient is an important parameter describing the long-term stability of the sensor. In order to sense the long-term reliability of the film, we placed the Bi_2_O_3_ sensing film treated with plasma treatments in a pH7 solution for 12 h to obtain the drift rate of the sensing film. The drift rate of the Bi_2_O_3_ sensing film without plasma was 23.58 mV/hr, and the drift rate of the sensing film with NH_3_ plasma after 1, 3, and 6 min were 20.7, 2.57, 15.09 mV/hr. It can be seen that the sensing film after 3 min of NH_3_ plasma had the lowest drift rate. This is because NH_3_ plasma could effectively passivate the defects, which allow ions to adhere, thereby inhibiting the diffusion of reactive ions and varying the reference voltage response. Therefore, the drift rate was reduced. Figure 8d shows the drift coefficient of the Bi_2_O_3_ sensing film after N_2_O plasma. The drift rate of Bi_2_O_3_ sensing film without plasma was 23.58 mV/hr, and the sensing film after N_2_O plasma for 1, 3, and 6 min were 2.45, 3.44, and 7.57 mV/hr. The sensing film of N_2_O plasma had the lowest drift rate in the sample with N_2_O plasma for 1 min.

## 4. Conclusions

Bi_2_O_3_ EIS sensing membranes in EIS structures were fabricated. To boost the sensing performance, NH_3_ and N_2_O plasma treatment were performed on the membranes. The results indicated that the sample treated with NH_3_ plasma for 3 min and the sample with N_2_O plasma treatment for 1 min had higher sensitivity than all the other conditions. Multiple material characterizations confirmed the enhancement of crystallization, and the removal of the defects may cause the improvements of the sensing behaviors owing to nitrogen passivation in the device. The plasma treatments could cause N atoms to incorporate into the bulks and the silicate could be transformed to well-crystallized films. Furthermore, plasma treatment could enhance grainization, which increased the density of the sensing surface sites, thereby boosting the sensing behaviors. Therefore, NH_3_ or N_2_O plasma treated-Bi_2_O_3_ membranes could reach the pH sensitivity around 60 mV/pH and show promise for future biomedical applications.

## Data Availability

Not applicable.
